# New method to determine proton trajectories in the equatorial plane of a dipole magnetic field

**DOI:** 10.1186/s40064-015-0917-7

**Published:** 2015-03-14

**Authors:** Damaschin Ioanoviciu

**Affiliations:** Physics Faculty and Institute of Doctoral Studies, Babes-Bolyai University, M. Kogalniceanu Str.1, 400084 Cluj-Napoca, Romania

**Keywords:** Proton orbits, Van Allen belts, Trapped protons, Dipole magnetic field, Stability condition

## Abstract

A parametric description of proton trajectories in the equatorial plane of Earth’s dipole magnetic field has been derived. The exact expression of the angular coordinate contains an integral to be performed numerically. The radial coordinate results from the initial conditions by basic mathematical operations and by using trigonometric functions. With the approximate angular coordinate formula, applicable for a wide variety of cases of protons trapped in Earth’s radiation belts, no numerical integration is needed. The results of exact and approximate expressions were compared for a specific case and small differences were found.

## Background

The differential equations of the motion of charged particles in a magnetic dipole field, as approximation of the Earth’s magnetic field, were derived already by Stőrmer (1907). Their solutions were obtained only numerically in an attempt to explain the behaviour of aurora borealis. The interest for charged particles trapped inside the dipole magnetic field increased by the discovery of Van Allen belts. A deep discussion of this topic can be found in Elliot (1963). Alfven (1950) developed an approximation splitting particle motion into guiding centre motion and gyration. The method has been enriched by Northrop (1961). Detailed studies of the motion of charged particles in the meridian plane of magnetic dipole fields are due to Markellos et al. (1978a) and (Markellos et al. 1978b).

The specific case of charged particle motion in the equatorial plane of the dipole magnet allowed De Vogelaere (1950) to obtain general periodic solutions of a Hill type differential equation from two numerically found particular solutions. Dragt (1964) in a comprehensive paper, describes a power series solution originating from a double power series solution of Stőrmer (1955). Graef and Kusaka (1938) obtained solutions for the motion in that plane in terms of elliptic integrals of first kind. These include a step of numerical calculation, no matter if the elliptic integral is obtained by a subroutine of the trajectory determining programme or if its value is picked up from tables.

Here parametric expressions of the proton coordinates in the equatorial plane were derived. Both exact radial and approximated angular coordinate expressions are strictly speaking, analytical formulas including only basic arithmetic operations and trigonometric functions. A prescribed accuracy for the angular coordinate is obtained from an exact formula by numerical integration, performed with the desired degree of precision.

## Results and discussion

### Radial coordinate-exact expression

**Figure 1:**
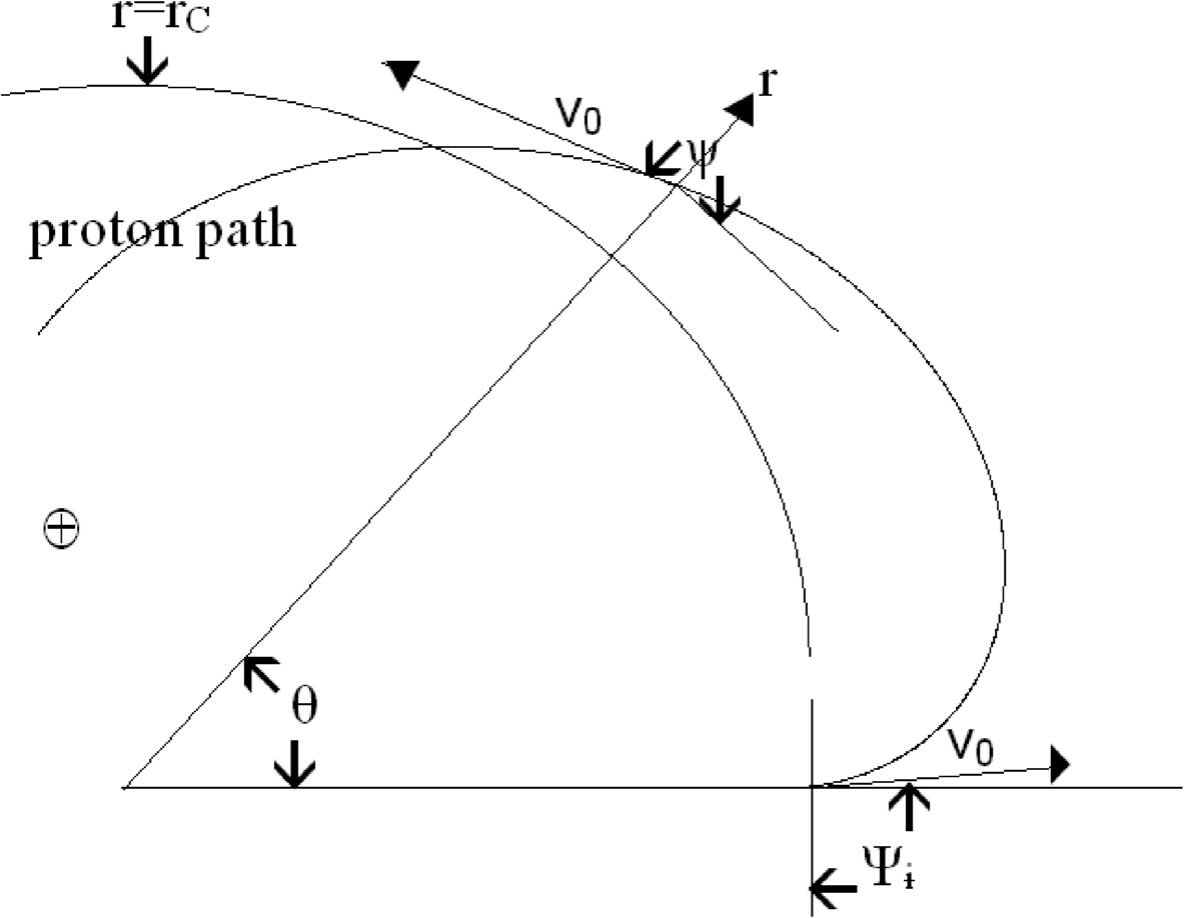
**Proton trajectory in the dipole magnet equatorial plane.** Defined are the radial r, and angular θ, coordinates (polar coordinates), the parameter ψ, the initial quantities v_0_, r_C_ and ψ_i_ = π/2.

We use a cylindrical coordinate system, Figure 1, with the polar coordinates r, θ in the equatorial plane of the dipole magnetic field (that of paper). Therefore, the z axis is directed along the dipole, the origin being located in its middle. A proton, moving with the velocity v_0_ in the equatorial plane will be pushed towards the origin if the magnetic field lines are directed to enter inside the paper, from outside the Earth’s body. In other words, we are looking at the figure from the Earth’s North magnetic pole. To determine the coordinate r we use two motion constants: the velocity v_0_ and the angular momentum, Eq. 3.35 from Miyamoto (2000), p.26.

1 $$ {v}^2={r}^2+{r}^2{\theta}^2={v}_0^2 $$

2 $$ m{r}^2\theta +qr{A}_{\theta }={c}_t $$

“Point” means derivative with respect to the time.

c_t_ is a constant. q and m are the proton charge and mass respectively. A_θ_ is the only non vanishing vector potential component. For z = 0 this is:

3 $$ {A}_{\theta }=\mu /{r}^2 $$

with μ the Earth’s magnetic dipole moment.

Next we use Richardson’s (1947) parameter ψ, the angle between the velocity and the theta velocity component, by the substitution v_θ_ = v_0_cosψ, earlier applied to describe ion paths in “wedge” magnetic fields and ion optical studies, see also Ioanoviciu (1989).

After substitution of A_θ_ and $r\theta$ in (2) we have:

4 $$ m{r}^2\theta +q\mu /r={c}_t $$

next we put:

5 $$ r\theta =-{v}_0 \cos \psi $$

and we obtain:

6 $$ \mu q/r-m{v}_0r \cos \psi ={c}_t $$

The constant c_t_ results from the initial conditions:

7 $$ r = {r}_i,\kern0.62em r\theta =-{v}_0 \cos {\psi}_i $$

when the parameter takes the value ψ = ψ_i_.

8 $$ \mu q/{r}_i-m{v}_0{r}_i \cos {\psi}_i={c}_t $$

Eliminating c_l_, between (6) and (8), next substituting r by ρ_i_ = r/ri we have:

9 $$ {\rho}_i^2 \cos \psi -{\rho}_i \cos {\psi}_i+{\eta}_i\left({\rho}_i-1\right)=0 $$

where:

10 $$ {\eta}_i=\mu q/\left(m{v}_0{r}_i^2\right) $$

Two solutions for ρ_i_ result:

11 $$ {\rho}_i=\left[ \cos {\psi}_i-{\eta}_i\pm \sqrt{{\left({\eta}_i- \cos {\psi}_i\right)}^2+4{\eta}_i \cos \psi}\right]/\left(2 \cos \psi \right) $$

Only the solution with “plus” sign offers physically acceptable values.

From the above expressions we obtain the maxima and minima of the proton radial coordinate by derivation with respect to ψ. As ρ_i_ is a function ρ_i_(cosψ) the derivative is

dρ_i_/dψ = dρ_i_/d(cosψ)(−sinψ) It vanishes for ψ = π and ψ = 2π values (if we limit to the first loop) that substituted in the solutions for ρ_i_ give the searched extreme values:

12 $$ {\rho}_i=\left[{\eta}_i- \cos {\psi}_i-\sqrt{{\left({\eta}_i- \cos {\psi}_i\right)}^2-4{\eta}_i}\right]/2 $$

for cosψ = −1 ,

and

13 $$ {\rho}_i=\left[ \cos {\psi}_i-{\eta}_i+\sqrt{{\left({\eta}_i- \cos {\psi}_i\right)}^2+4{\eta}_i}\right]/2 $$

for cosψ = 1

Further radial coordinate expression simplification can be obtained if we select the observation starting point for ψ_i_ = π/2:

14 $$ \rho =\left[\sqrt{\eta \left(\eta +4 \cos \psi \right)}-\eta \right]/\left(2 \cos \psi \right) $$

Here the index “i” has been removed.

Now ρ = r/r_C_, η = μq/(p_r_r_C_^2^) with r_C_ the radial distance for ψ_i_ = π/2, p_r_ the relativistic particle momentum ${p}_r={m}_0{v}_0/\sqrt{1-{\beta}^2},$

The extreme values of ρ are then:

15 $$ \rho =\left[\eta -\sqrt[]{\eta \left(\eta -4\right)}\right]/2 $$

16 $$ \rho =\left[\sqrt{\eta \left(\eta +4\right)}-\eta \right]/2 $$

We can use the simplified expressions by looking for an equivalent equation connecting r_i_ and η_i_ to r_C_ and η. By equating the maximum and the minimum of the equation (12), (13) to those of eq. (15), (16) we obtain the following equivalence:

The eq. (11) with “minus” sign, is identical with (14) if:.

17 $$ {r}_C={\eta}_i{r}_i/\left({\eta}_i- \cos {\psi}_i\right) $$

and

18 $$ \eta ={\left({\eta}_i- \cos {\psi}_i\right)}^2/{\eta}_i $$

The stability condition for the proton trajectories results from the equation of ρ. The condition to keep the charged particle trapped inside the dipole magnetic field: ρ must be real. This happens always if:

19 $$ \eta >4\ge 4 \cos \psi $$

or by detailing η:

20 $$ {p}_r<\mu q/\left(4{r}_C^2\right) $$

### Angular exact coordinate expression

The angular coordinate, θ is obtained by an integration to be performed numerically with the desired accuracy degree.

21 $$ d\theta =\left(d\theta /dr\right)dr=\frac{\theta }{\overset{.}{r}}dr=\left(-{v}_0 \cos \psi /r\right)/\left({v}_0 \sin \psi \right)dr $$

Finally we integrate over ψ from the beginning at ψ = π/2 to the current value of ψ:

22 $$ \theta =-{\displaystyle \int \cos \psi \left(\rho \sin \psi \right)d\rho =\frac{1}{2}{\displaystyle \underset{\pi /2}{\overset{\psi }{\int }}\left[\frac{\sqrt{\eta }}{\sqrt{\eta +4 \cos \psi }}-1\right]d\psi }} $$

### Approximating proton coordinates

Let’s estimate the η values for the Van Allen belts. For the Earth, dipole moment μ_E_ = 7.906×10^15^ T.m^3^, equatorial radius R_E_ = 6.378×10^6^m , the protons of 10÷100 MeV, moving inside the inner belt concentrated around 1.5R_E,_ have η = 188.536 to 58.247 (Hess 1962; Fiandrini et *al*. 2004).

For the protons of 1 MeV located between 2.5 and 8R_E_ (second belt) the values of η=215.147 and 21.01 while for those of 0.065 MeV η=844.087 and 82.43.

The enumerated η values suggest when the integral giving θ (as r values result straightforward) can be obtained with accuracy. We take the equation (9) and divide it by η. Accounting for 1/η as small quantity, we substitute 1/η = α and ρ = 1 + ε , both α and ε being assumed to be small quantities. Then we obtain:

23 $$ {\rho}^2/\eta -\rho \left( \cos {\psi}_i/\eta -1\right)/ \cos \psi -1/ \cos \psi =0 $$

That after the substitutions gives:

24 $$ \left[\alpha \cos \psi {\left(1+\varepsilon \right)}^2+\varepsilon \right]/\alpha =0 $$

By successive approximation, we obtain the following expression for, ε after the third approximation step:

25 $$ \varepsilon =-\alpha \cos \psi \left(1-2\alpha \cos \psi +5{\alpha}^2{ \cos}^2\psi \right) $$

ρ obtained as 1 + ε has been substituted in the expression of dθ :

26 $$ d\theta =- \cos \psi /\left[\left(1+\varepsilon \right) \sin \psi \right]d\varepsilon $$

As dε has the form:

27 $$ d\varepsilon =\alpha \sin \psi \left(1-4\alpha \cos \psi +15{\alpha}^2{ \cos}^2\psi \right)d\psi $$

to obtain θ we have to integrate ψ from π/2 to the current ψ value.

28 $$ d\theta =-\alpha \cos \psi \left(1-3\alpha \cos \psi +10{\alpha}^2{ \cos}^2\psi \right)d\psi $$

After integration it results:

29 $$ \theta =\alpha \left(1- \sin \psi \right)+\left(3/2\right){\alpha}^2\left( \sin \psi \cos \psi +\psi -\pi /2\right)-\left(10/3\right){\alpha}^3\left( \sin \psi { \cos}^2\psi +2 \sin \psi -2\right) $$

### Application to a specific case

The case of a trapped 60 MeV proton oscillating around the characteristic radial distance of 1.5 Earth radii was considered. The motion characterizing parameter results then to be η = 75.97. The exact coordinates ρ and θ, as well as the relative difference between the exact and the approximated angular coordinates (θ-θ_app_)/θ in percents were presented, for a complete loop, in Table 1. The error is of the order of 0.002% to 0.12%.

**Table 1:** **First loop of a 60 MeV energy proton with the basic radial distance**

**ρ**	**θ in radians**	**( θ-θ**_**app**_ **)/θ %**
1.000000	0	
0.99674778	2.046283×10^−3^	0.1213
0.991148	5.138577×10^−3^	0.0476
0.987785	1.048920×10^−2^	0.0209
0.987394	1.203098×10^−2^	0.0173
0.990058	6.310101×10^−3^	0.0381
0.995200	2.626267×10^−2^	0.0050
1.001655	2.707027×10^−2^	0.0048
1.007859	2.462010×10^−2^	0.0052
1.0122026	1.941538×10^−2^	0.0055
1.013494	1.272709×10^−2^	0.0045
1.011368	6.208492×10^−3^	0.0021
1.006424	2.552444×10^−2^	0.0050
1.000000	1.635483×10^−3^	0.1150

r_C_=1.5×6.378×10^6^m, η=75.97.

Parametric calculated exact coordinates ρ=r/r_C_ and θ, as well as the error, (θ-θ_app_)/θ by using the approximate angular coordinate formula θ_app_, were tabulated.

**Figure 2:**
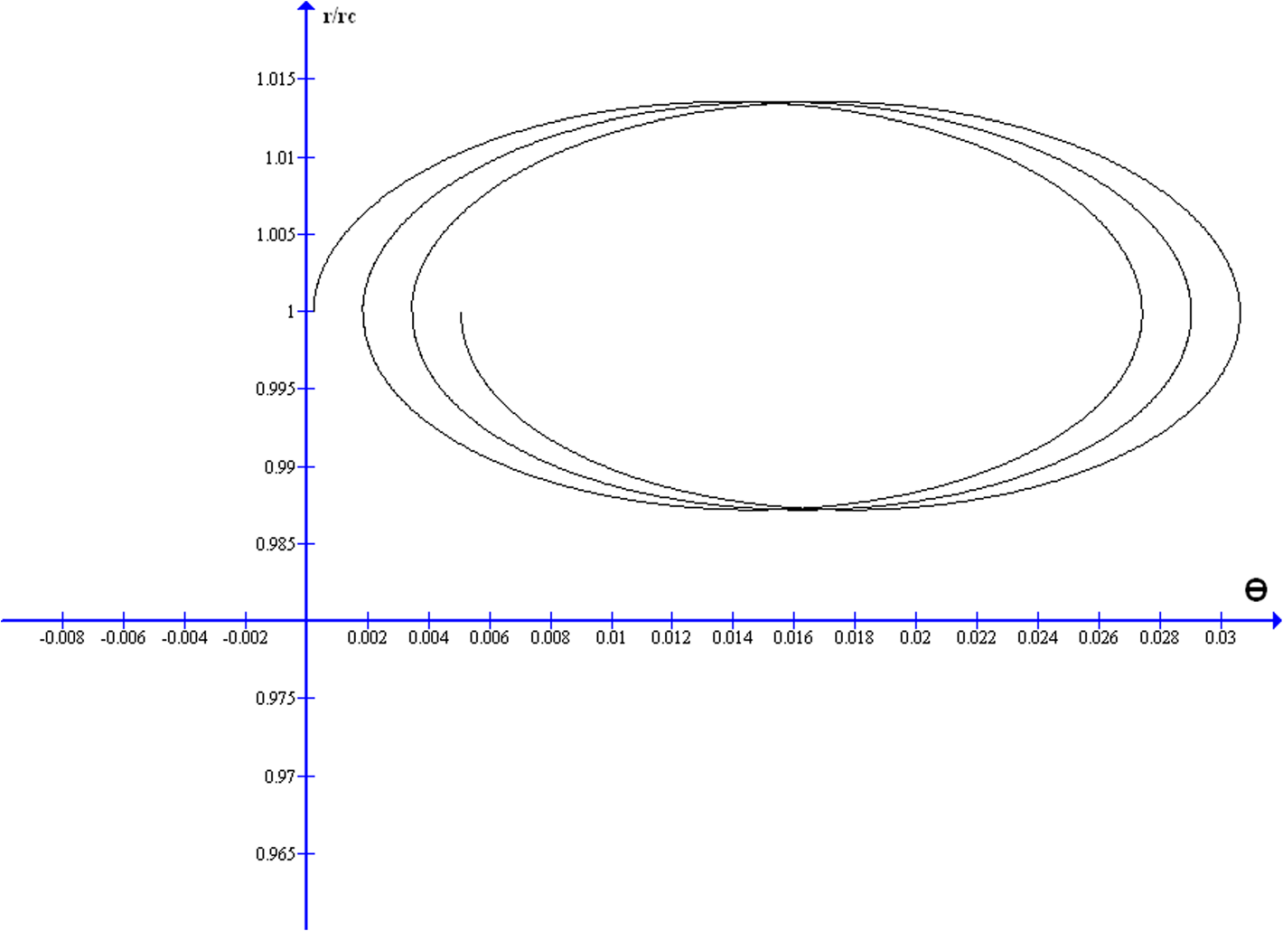
**Three loops of a 60 MeV proton inside the Earth’s dipole magnetic field equatorial plane.** The ratio ρ = r/r_C_ is represented in ordinate as function of the angle θ as abscissa. The three loops correspond to ψ parameter variation intervals: π/2 to 5π.2 the first, 5π/2 to 9π/2 the second and 9π/2 to 13π/2 the third respectively.

In Figure 2 the reduced radial distance r/r_C_ exact values were represented as function of θ_app_ in abscissa, for three consecutive complete loops, corresponding to the intervals, ψ = π/2 to 5π/2, ψ = 5π/2 to 9π/2 and ψ = 9π/2 to 13π/2 respectively.

## Conclusion

The coordinates of the protons moving in the Earth’s equatorial plane were derived as functions of a parameter. The use of the exact expressions assumes only basic operations and trigonometric functions to be involved for the radial coordinate calculation, while for the angular coordinate a numerical integration is necessary. Combining the exact radial formula with the approximated angular coordinate expression, an entirely analytic set of formulas has been obtained. The accuracy of this description has been shown on an illustrative example. The amazing simplicity of the coordinate expressions suggests possible developments by accounting for field perturbations.
